# *In vivo* Downregulation of MHC Class I Molecules by HCMV Occurs During All Phases of Viral Replication but Is Not Always Complete

**DOI:** 10.3389/fcimb.2020.00283

**Published:** 2020-06-12

**Authors:** Florin Gabor, Gerhard Jahn, Daniel D. Sedmak, Christian Sinzger

**Affiliations:** ^1^Institute of Medical Virology, University of Tübingen, Tübingen, Germany; ^2^Institute of Pathology, The Ohio State University, Columbus, OH, United States; ^3^Institute of Virology, Ulm University Medical Center, Ulm, Germany

**Keywords:** cytomegalovirus, immune evasion, immunohistochemistry, MHC class I, antigen presentation

## Abstract

Based on cell culture data, MHC class I downregulation by HCMV on infected cells has been suggested as a means of immune evasion by this virus. In order to address this issue *in vivo*, an immunohistochemical analysis of tissue sections from biopsy and autopsy materials of HCMV infected organs was performed. HCMV antigens from the immediate early, early, and late phase of viral replication, and cellular MHC class I molecules were detected simultaneously or in serial sections by immuno-peroxidase and immuno-alkaline phosphatase techniques. Investigated organs included lung, gastrointestinal tract, and placenta. Colocalization of MHC molecules with sites of viral replication as well as MHC expression in individual infected cells were analyzed. To detect immune effector cells at sites of viral replication, leukocytes, CD8+ lymphocytes, and HCMV antigens were stained in serial sections. While strong MHC class I expression was detected in the cells surrounding infected cells, it appeared downregulated in the majority of infected cells themselves, particularly in the late replication phase. Despite significantly reduced MHC class I signals on infected cells, sites of infection were infiltrated by inflammatory cells that consisted predominantly of CD8+ lymphocytes. The extent of inflammatory infiltrates was negatively correlated with the extent of HCMV infected cells. Taken together, our findings indicate that HCMV can downmodulate MHC class I expression *in vivo*, whereas cytokines originating from infiltrating immune effector cells probably up regulates MHC class I expression in noninfected bystander cells. The presence of cytotoxic lymphocytes in close contact to infected cells may reflect control of viral spread by these cells despite MHC class I downmodulation.

## Introduction

Human cytomegalovirus (HCMV), also known as human betaherpesvirus 5, is distributed worldwide with seroprevalence rates between 45 and 100% (Cannon et al., 2010). After the primary infection, HCMV persists for the lifetime in a latent state from which it can be reactivated, shed in body secretions and thereby transmitted to other hosts. It is remarkable that HCMV can regularly establish a persistent infection although it expresses more than a 100 proteins that can be targeted by B-cell and T cell responses (Hengel et al., 1998). Indeed, the virus dedicates a great part of its genome to counteract various effectors of the immune system and thus escape from elimination (Loenen et al., 2001; Wilkinson et al., 2008; Jackson et al., 2011; Trilling et al., 2012; Corrales-Aguilar et al., 2014; Goodier et al., 2018). The result is an intricate balance between host defense and viral immune evasion mechanisms that allow the virus to spread successfully in the human population while the host usually stays undamaged.

In immunocompetent hosts, symptoms during the primary infection are typically mild, and during the latent phase the virus is well-controlled by a humoral and cellular immune response that specifically targets many of the viral proteins (Sylwester et al., 2005; McCormick and Mocarski, 2015; Jackson et al., 2019). Among cytotoxic T cell responses, cells directed against the non-structural immediate early antigen (pUL122/123), and the structural tegument protein pp65 (pUL83) predominate and can account for up to 20 and 27 % of the total CD8+ T cells in a normal host in the absence of apparent infection or reactivation (Khan et al., 2007). Such T cells are not only numerous but most probably effective in controlling the virus, as donor CTLs directed against those epitopes proved to protect recipients in a post-transplant situation when applied for adoptive immune transfer (Riddell et al., 1992; Feuchtinger et al., 2010; Jackson et al., 2014; Neuenhahn et al., 2017; Kaeuferle et al., 2019).

Immune evasion mechanisms have been attributed to HCMV that can attenuate various immune effectors, including interferons, NK cells, antibodies, and T cells (Wilkinson et al., 2008; Jackson et al., 2011; Trilling et al., 2012; Corrales-Aguilar et al., 2014; Goodier et al., 2018). In particular, as cytotoxic T cell responses are regarded crucial for the control of HCMV (Portela et al., 1995; Kaeuferle et al., 2019), viral proteins directed against MHC class I restricted antigen presentation have been extensively studied. One of these proteins is already expressed immediately after viral entry into the host cell (Ahn et al., 1996; Jones et al., 1996). This protein is encoded by the viral gene US3 and prevents intracellular transport of MHC class I molecules toward the cell surface. During the early phase of replication this function is further corroborated by two proteins, encoded by the viral genes US2 and US11, that dislocate newly synthesized MHC class I molecules from the ER to the cytoplasm, where they are then targeted for degradation by the proteasome (Jones et al., 1995, 1996; Wiertz et al., 1996). And finally during the late phase of infection, the protein encoded by US6 adds a third mechanism by blocking the translocation of peptides to the ER via the transporter associated with antigen presentation (TAP), thereby preventing peptide loading onto MHC class I molecules that may have escaped from degradation by US2 and US11 (Hengel et al., 1996, 1997; Ahn et al., 1997; Lehner et al., 1997).

At a first glance it may appear surprising why the virus encodes two proteins, pUS2, and pUS11, that serve the same function, which is targeting MHC class I molecules for degradation by dislocating them into the cytoplasm. Slight differences in their mode of action or their efficacy in different cell types or tissues may provide an explanation (Gewurz et al., 2001; Rehm et al., 2002; Frascaroli et al., 2018). For US2 an effect was also reported on MHC class II molecules (Tomazin et al., 1999; Hegde and Johnson, 2003), but profound downmodulation of class II molecules on the cell surface also occurs in the absence of US2 and the genes involved are mostly unknown (Cebulla et al., 2002; Lučin et al., 2015). Importantly, not all MHC class I molecules are degraded by these proteins. HLA-C and HLA-G are apparently resistant to pUS2 and pUS11 (Schust et al., 1998), indicating that the host can counteract the effect of US22 and US11 to some extent by its polymorphism in HLA genes. Furthermore, the action of cytokines like type I and II interferons and tumor necrosis factor alpha, released from inflammatory cells infiltrating into infected tissues, can also rescue some degree of antigen presentation by a general upregulation of MHC class I expression (Benz and Hengel, 2000).

To date, we can only speculate about the net effect of these complex and antagonistic interactions between the viral and host factors on MHC class I expression in organs that are relevant in the pathogenesis of HCMV infections. While downmodulation of MHC class II molecules has already been shown in HCMV-infected cells in lung tissue by immunohistochemical means (Ng-Bautista and Sedmak, 1995), similar *in vivo* data concerning MHC class I downmodulation in relevant target organs of HCMV-associated disease, like lung, and colon, are still missing. Beside such target organs of HCMV-associated disease, placental tissue may be particularly informative in this regard, because it is regularly infected in cases of congenital HCMV infection and all immune mechanisms are in principle unaffected in the developing fetus. Therefore, the situation in the placenta may be closer to the situation in the normal host than tissue specimens from severely immunocompromised patients.

Taken together, a plethora of data demonstrates the capability of HCMV to downregulate MHC class I molecules and thereby evade the recognition by cytotoxic T cells in cell culture systems whereas, on the other hand, HCMV infections are known to be well-controlled in immunocompetent hosts and HCMV-specific cytotoxic lymphocytes may contribute to this control as they could protect recipients from HCMV infections in adoptive transfer studies. Remarkably little data are available regarding MHC class I modulation and the consequences for a cytotoxic T-cell reaction *in vivo* in the complex situation of an infected tissue. To address this issue, we analyzed samples from HCMV-infected tissues by immunohistochemical means concerning the questions of whether evidence of MHC class I downregulation can be found *in vivo* in the different phases of viral replication and whether this might allow viral escape from the cytotoxic T cell response.

## Materials and Methods

### Tissue Sections

Formalin fixed, paraffin embedded tissues with light microscopic evidence of active HCMV infection were provided by the Cooperative Human Tissue Network, a National Cancer Institute supported resource. Other investigators may have received samples from these same tissue specimens. All placentas and lung biopsies, resections, or autopsies performed at The Ohio State University Medical Center within a three year period were searched for the diagnosis of CMV. The histology of ~10 cases found by the search was reviewed and those tissues that showed several CMV-infected cells per high power field were selected. In order to allow for a correlative analysis of virus infection, MHC class I expression and lymphocytic infiltrations in serial sections, the tissue blocks contained well-preserved tissue with a high number of HCMV-infected cells. To represent several typical clinical situations of HCMV replication, two lung and two colon tissues of immunocompromised patients and two placental tissues of congenitally infected children were chosen. Formalin-fixed paraffin-embedded tissues were sectioned at 3 μm, mounted on coated glass slides, and numbered to allow for correlative analyses of various antigens in serial sections.

### Antibodies

For detection of HCMV-infected cells in the different stages of viral replication, monoclonal antibodies (MAbs) against various viral proteins were used. MAb El3 (Biosoft) is directed against the non-structural immediate early antigens (pUL123 and pUL122) which are detectable throughout the replication cycle (Mazeron et al., 1992). MAb CCH2 (generously provided by B. Zweygberg Wirgart and L. Grillner, Stockholm, Sweden) is directed against the non-structural early 52 kDa DNA-binding protein (pUL44) and is detectable in the early and late phase of viral replication (Plachter et al., 1992). MAb XP1 (NCNL 03; Behringwerke), which is directed against the cytoplasmic tegument protein pp 150 (pUL32), was used to detect late-stage infected cells (Jahn et al., 1990).

For detection of MHC class I antigens, we used MAb HC10 (kindly provided by H. Ploegh) that was raised against free class I, HLA-B locus heavy chains (Stam et al., 1986), and reacts with HLA-B, and HLA-C heavy chains, and some HLA-A heavy chains (HLA-A10, HLA-A28, HLA-A29, HLA-A30, HLA-A31, HLA-A32, HLA-A33) (Stam et al., 1990). This antibody was chosen as it yields a clear staining pattern in formalin-fixed paraffin-embedded tissues that correlates well with the staining pattern of the pan-HLA-antibody W6/32 in frozen sections (Stam et al., 1986; Mattijssen et al., 1991).

For detection of lymphocytes, a combination of antibody clones 2B11 and PD7/26 (Dako) was used, which is directed against leukocyte common antigen (CD45). For identification of cytotoxic lymphocytes, antibody clone C8/144B (Dako) was used, which is directed against CD8.

### Immunohistochemical Staining

Tissue sections were deparaffinized and endogenous peroxidase was blocked by incubation with 0.6% hydrogen peroxide in methanol for 20 min at room temperature (RT). Tissue sections were then rehydrated with graded ethanol and digested with 0.1% protease (Merck, Darmstadt, Germany) in phosphate-buffered saline (PBS) for 5 min at RT.

For detection of HCMV early antigen, sections were pretreated with 0.1% protease type 24 (Sigma) in PBS for 5 min at RT. For detection of HCMV immediate early or late antigen, MHC class I molecules or leukocyte common antigen, sections were pretreated with antigen retrieval solution (Dako). For detection of CD8, sections were boiled with 1 mmol/l EDTA solution (pH = 8.0) in a pressure cooker. To prevent nonspecific binding of antibodies, rabbit non-immune serum was added for 20 min. Non-immune serum was then removed and tissue sections were subsequently incubated with primary antibodies, secondary antibodies, and staining reagents to visualize the antigen of interest.

For brown staining, tissue sections were first incubated with the primary antibody of choice for 90 min at RT, followed by incubation with biotinylated rabbit anti-mouse Ig-antibody (Dako) for 60 min at RT and lastly incubated with streptavidin-biotin-peroxidase complexes (Dako) for 20 min at RT. Sections were rinsed in PBS for 5 min after each incubation step. Staining was performed using 0.6% DAB in PBS.

For red staining, tissue sections were first incubated with the primary antibody of choice for 90 min at RT, followed by incubation with biotinylated rabbit anti-mouse Ig-antibody (Dako) for 60 min at RT and lastly incubated with streptavidin-biotin-alkaline phosphatase complexes (Dako) for 20 min at RT. Sections were rinsed in 0.05 mol/l Tris (pH = 7.6) for 5 min after each incubation step. Staining was performed using Fast Red TR/Naphthol AS-MX Tablets (Sigma).

Finally, tissue sections were counterstained with hematoxylin, mounted in glycerol gelatin, and analyzed with a Polyvar light microscope (Cambridge Instruments).

### Quantitative Analyses and Statistical Tests

Quantitative analyses of signal intensities were done using the “measure” function of ImageJ software (Schneider et al., 2012). Images of immunostained placental sections were taken with an AxioLab.A1 microscope (Zeiss) using an Axiocam 105 color camera. Using Fuji software (Schindelin et al., 2012), the stromal regions of individual villi were defined as regions of interest (ROI) and the brightness was measured as the mean gray level per pixel on a scale of 256 gray levels. In each image, empty parts without tissue were also measured for background correction. Using Excel software, signal intensities were calculated as the reduction of background brightness by the specific staining (brightness background—brightness ROI). The signal intensities of all “infected” villi were compared with the signal intensities of all “uninfected” villi, using the inbuilt *t*-test function (two-sample, unequal variance) of Excel.

Categorial variables were analyzed for independence by a chi-squared test using the inbuilt CHISQ.TEST function of Excel.

## Results

### MHC Class I Downregulation in Infected Cells

For several gene products of HCMV a clear MHC class I downmodulating effect has been demonstrated in cell culture systems both after transfection of isolated genes and in the viral context. On the other hand, inflammatory cytokines can upregulate MHC class I molecules. To investigate whether HCMV infection results in downmodulation of MHC class I expression in the complex situation of an infected tissue, we analyzed sections from formalin fixed paraffin-embedded infected lung, colon, and placental tissue by immunohistochemical double labeling for viral antigen and MHC class I heavy chains.

**Figure 1:**
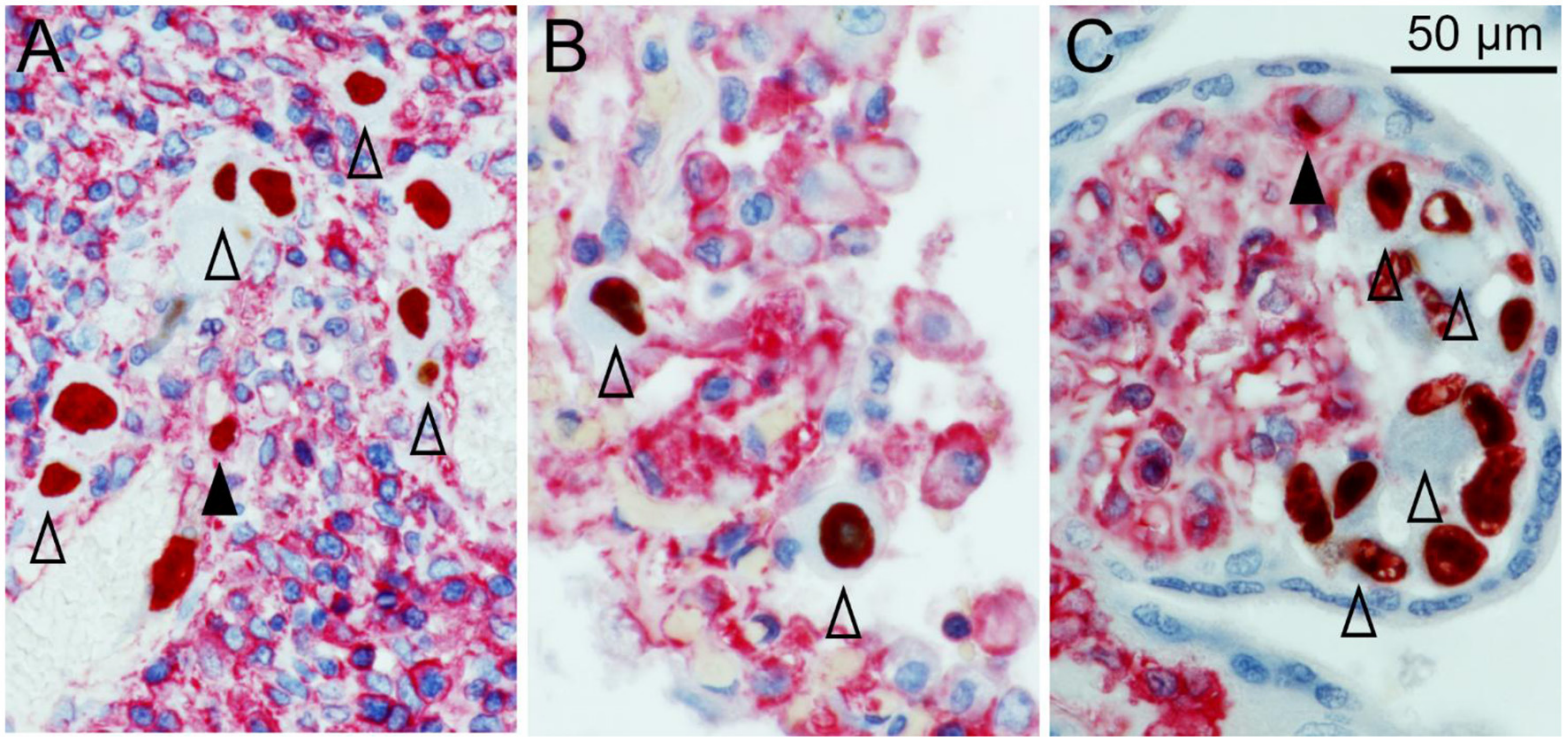
MHC class I downregulation in infected cells. HCMV immediate early antigen (brown, nuclear) and MHC I molecules (red, cytoplasm and cell surface) were detected by immunohistochemical staining in tissue sections from HCMV-infected colon **(A)**, lung **(B)**, and placenta **(C)**. Nuclei were counterstained with hematoxylin (blue). Examples of infected cells without detectable MHC I signals are indicated by open arrowheads. Infected cells with detectable MHC I signals are indicated by filled arrowheads. The scale bar is valid for all panels.

After deparaffination, blocking of endogenous peroxidase, rehydration, and antigen retrieval, the sections were first stained for viral immediate early antigen by an indirect immunoperoxidase technique resulting in brown nuclear signals. MHC class I molecules were then detected by an indirect alkaline phosphatase technique, resulting in a red cytoplasmic, and surface staining pattern. In all three tissues HCMV-infected cells were clearly distinguished from the surrounding non-infected cells by a remarkable lack of MHC class I signals. At least for those HLA alleles detected by the applied HC10 antibody (all HLA-B and HLA-C alleles and several HLA-A alleles) this indicates a strong downmodulating effect of HCMV infection. While in most infected cells no red signal at all could be discriminated, a minority of infected cells showed weak to normal signals as compared to adjacent cells, indicating that MHC class I downmodulation occurred later than immediate early viral gene expression and/or was not always complete (Figure 1). These cells were typically smaller than those that lacked MHC class I signals, suggesting that they might be in an earlier stage of viral replication.

**Figure 2:**
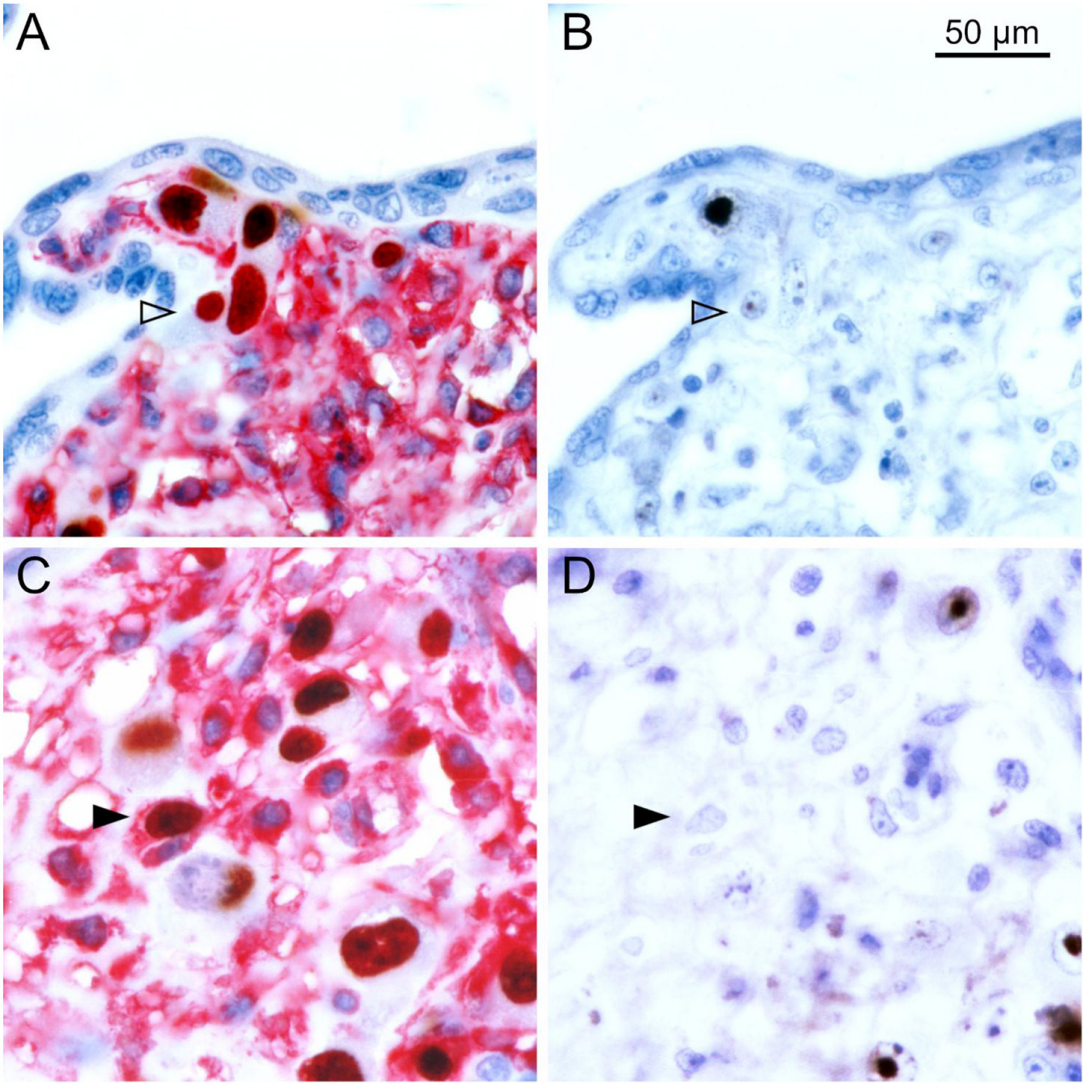
MHC class I expression during the immediate early stage of infection. Serial sections from HCMV-infected placenta tissue were analyzed by immunohistochemical staining regarding the association of MHC class I expression with the viral replication phase. **(A,C)** Viral immediate early antigen (brown, nuclear) and MHC I molecules (red, cytoplasm and cell surface) were detected by immuno-peroxidase and immuno-alkaline phosphatase techniques, respectively. **(B,D)** Viral early antigen (brown, nuclear) was detected by immuno-peroxidase technique. Nuclei were counterstained with hematoxylin (blue). Examples of cells in the immediate early phase (no early antigen detectable) with and without detectable MHC I signals are indicated by filled and open arrowheads, respectively. The scale bar is valid for all panels.

**Figure 3:**
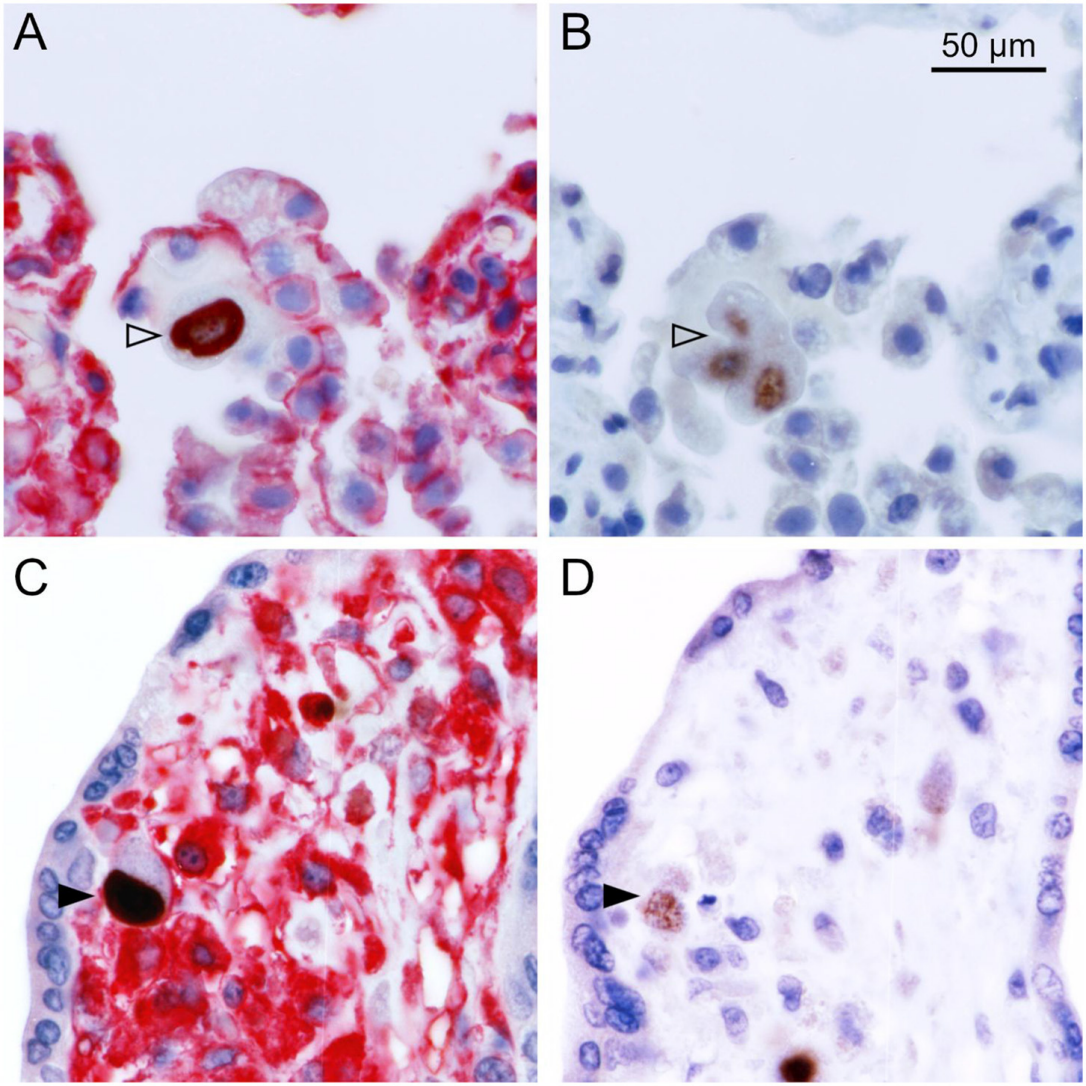
MHC class I expression during the late stage of infection. Serial sections from HCMV-infected lung **(A,B)** or placental **(C,D)** tissue were analyzed by immunohistochemical staining regarding the association of MHC class I expression with the viral replication phase. **(A,C)** Viral immediate early antigen (brown, nuclear) and MHC I molecules (red, cytoplasm, and cell surface) were detected by immuno-peroxidase and immuno-alkaline phosphatase techniques, respectively. **(B,D)** Viral late antigen (brown, nuclear) was detected by immuno-peroxidase technique. Nuclei were counterstained with hematoxylin (blue). Examples of cells in the late phase (both immediate early and late antigens detectable) with and without detectable MHC I signals are indicated by filled and open arrowheads, respectively. The scale bar is valid for all panels.

To differentiate the stages of HCMV replication in infected cells, we applied antibodies directed against immediate early and late viral proteins to serial sections of the tissues. The first section was immunoperoxidase-stained for an early viral nuclear protein, the second was stained by double labeling or immediate early antigen and MHC class I as described before, and the third section was immunoperoxidase-stained for a late viral nuclear protein. All infected cells will display immediate early antigen, those that have proceeded beyond the immediate early phase will display early antigen in addition and only those that entered the late productive phase of viral replication will display the late antigen. This correlative analysis of adjacent sections showed that MHC class I signals were detectable in most cells in the immediate early phase of replication but a few of these cells lacked MHC class I signals already at this replication phase (Figure 2). Cells in the late stage of infection always lacked MHC class I signals in the cytoplasm (Figure 3), but some of them showed a red margin, indicating some residual amount of MHC class I molecules at the surface of these cells (Figures 3C,D).

Taken together, this first set of experiments showed that clear signs of MHC class I downmodulation can be detected in various HCMV-infected tissues. This occurs principally in the late phase. A subset of infected cells in each stage of infection continued to show HLA expression, albeit at lower levels as demonstrated by a reduction in the intensity of staining.

### MHC Class I Expression and Immune Response in the Surrounding of Infected Cells

**Figure 4:**
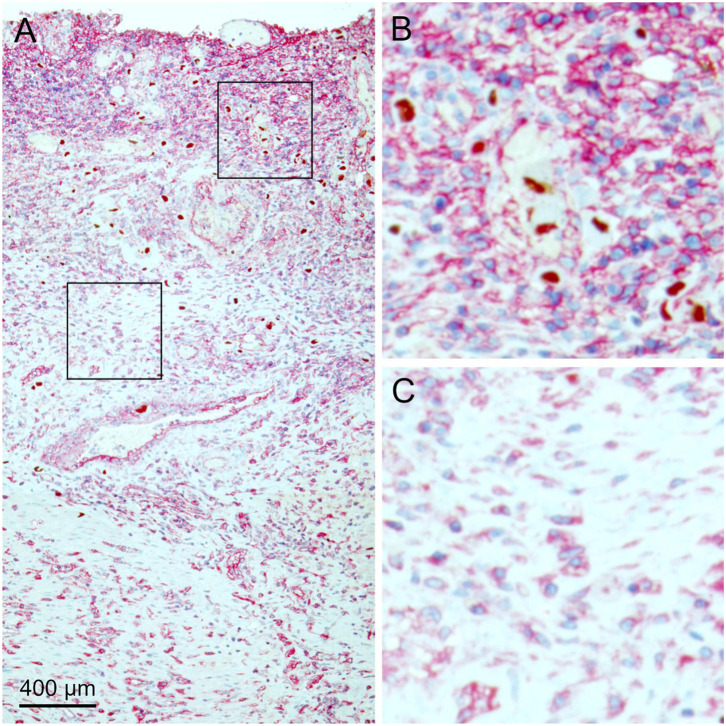
MHC class I expression in infected tissue. HCMV immediate early antigen (brown, nuclear) and MHC I molecules (red, cytoplasm, and cell surface) were detected by immunohistochemical staining in tissue sections from HCMV-infected colon tissue. Nuclei were counterstained with hematoxylin (blue). **(A)** The overview at low magnification shows that MHC class I expression is not homogenous throughout the tissue section. Areas with high **(B)** or low **(C)** MHC I signal intensity are enlarged, which represent the finding that the density of HCMV-infected cells was higher in the former.

During the analysis of tissue sections that were double stained for infected cells and MHC class I molecules, it became obvious that the expression of MHC class I molecules was not homogenous throughout the tissue sections as there were areas with higher and lower signal intensity (Figure 4A). A closer look suggested that increased MHC class I staining occurred at some but not all sites of infection, which seemed to correlate with the degree of inflammatory infiltration. In contrast, uninfected areas in the specimen consistently showed lower intensity of the red signal (Figures 4B,C).

To test whether this impression was true, we analyzed the placental specimen more in detail. Placenta was chosen, because it consists of numerous separated villi that could be easily distinguished in the tissue sections and counted as individual “events.” To exclude that the initial immune peroxidase staining of viral immediate early antigen interfered with the subsequent immuno-alkaline phosphatase staining of MHC class I molecules in a way that higher signal intensity was falsely amplified, we reversed the order of the staining steps.

**Figure 5:**
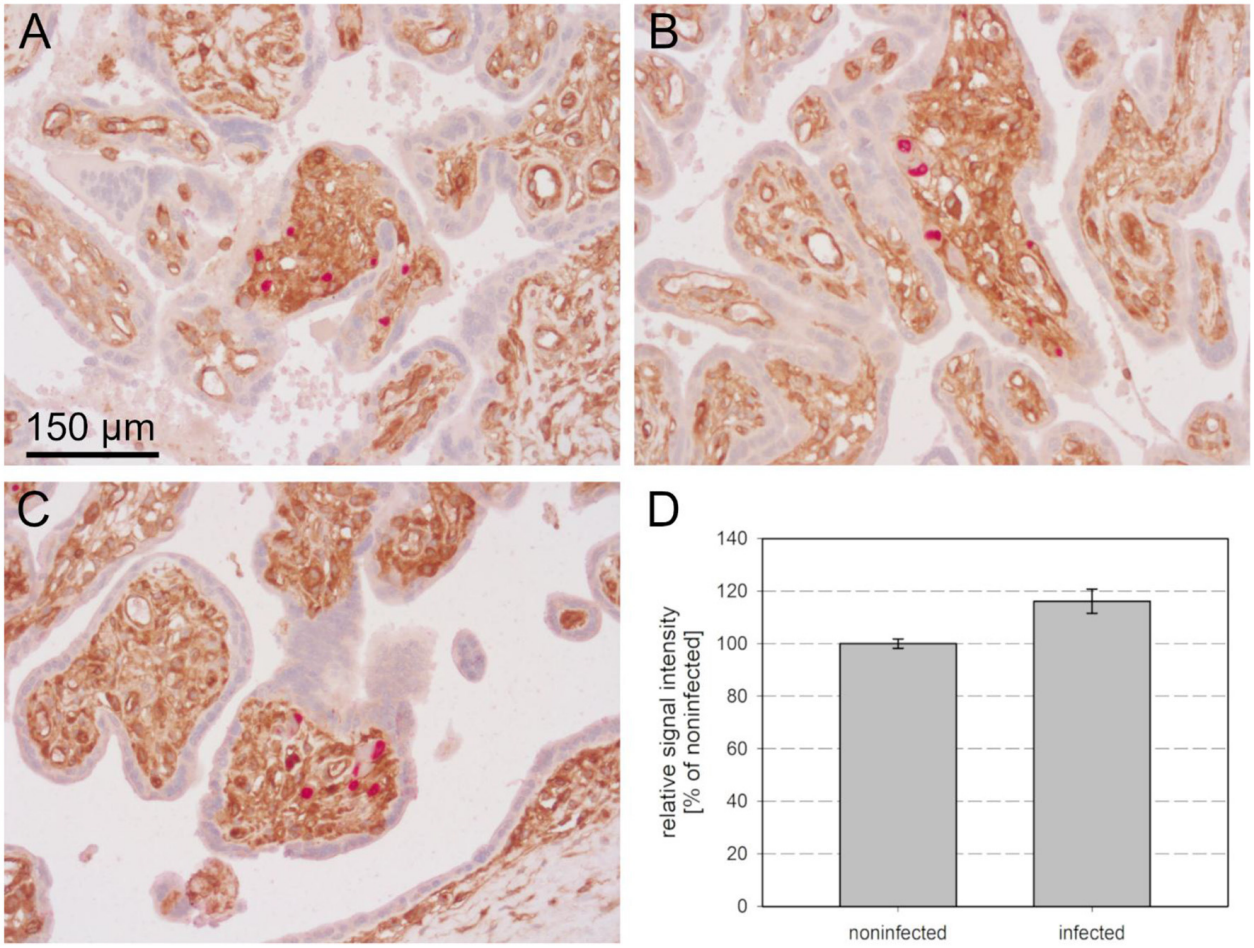
MHC class I expression in infected placental tissue. MHC I molecules (brown, cytoplasm and cell surface) and HCMV immediate early antigen (red, nuclear) were detected by immunohistochemical staining in tissue sections from placental tissue. Nuclei were counterstained with hematoxylin (blue). Most of the villi with focal infection showed stronger MHC class I signals than uninfected villi in the surrounding **(A,B)**, whereas intensity in uninfected villi was equally strong in a minority of sites **(C)**. The scale bar is valid for all panels. The mean signal intensity in the stroma of villi with focal infection was elevated as compared to the mean of HCMV-negative villi **(D)**.

After deparaffination, blocking of endogenous peroxidase, rehydration, and antigen retrieval, the sections were first stained for MHC class I molecules by an indirect immunoperoxidase technique resulting in brown cytoplasmic and surface signals. Viral immediate early antigen was then detected by an indirect alkaline phosphatase technique, resulting in a red nuclear staining and nuclei were counterstained with hematoxylin. The complete section was then systematically scanned for villi that showed foci of infected cells and these sites were photo-documented. The images revealed that all 17 villi which contained foci of infected cells also showed strong MHC class I expression in the non-infected stromal cells (Figures 5A–C). Among the 135 villi without infection, a fraction also had strong MHC class I expression in the stroma while in others the staining signals were less prominent. When mean signal intensities in the stroma of HCMV-positive villi were compared with the surrounding HCMV-negative villi in a quantitative analysis, a significant increase of 17 % was found in HCMV positive villi (*p* = 0.004), corroborating the initial impression of elevated MHC class I expression at sites of infection (Figure 5D).

**Figure 6:**
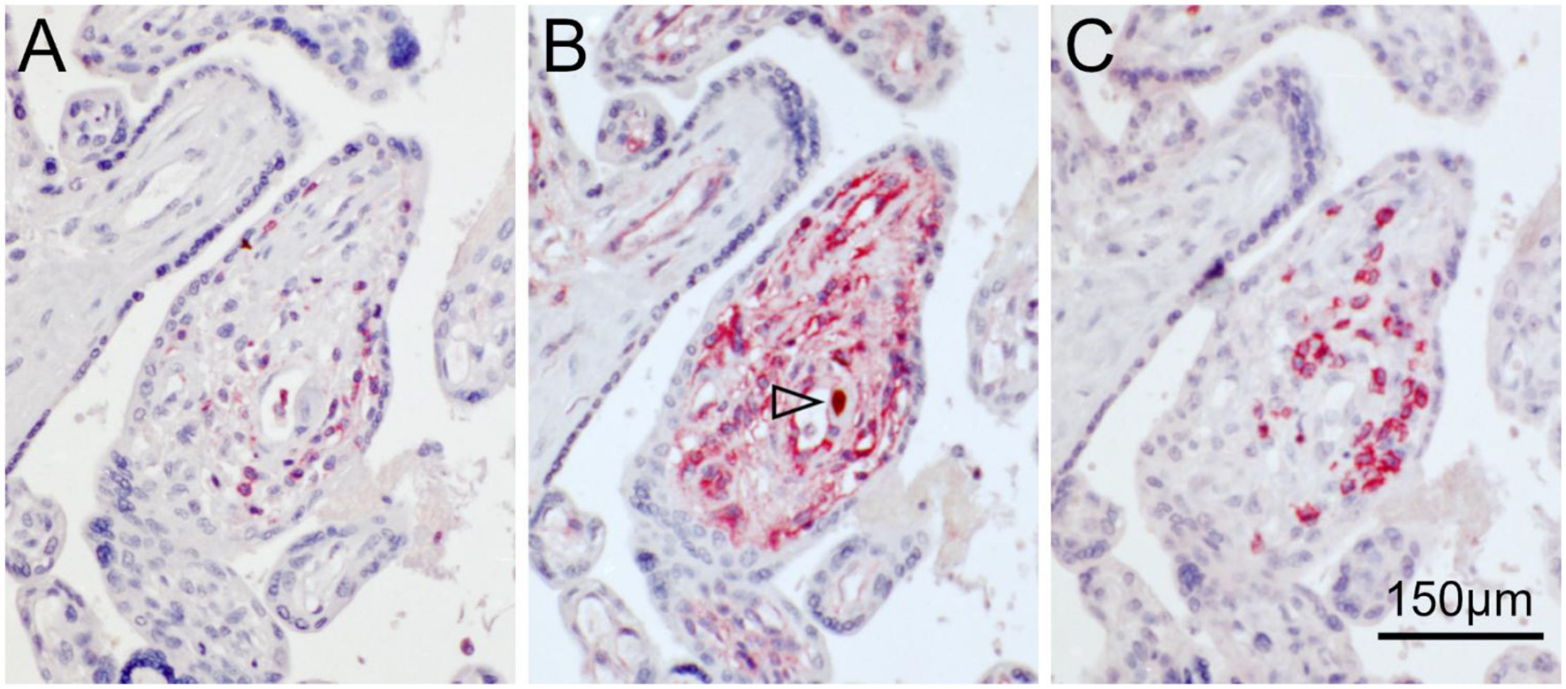
Colocalization of HCMV, MHC class I molecules and lymphocytes in placental tissue. Serial sections from HCMV-infected placenta tissue were analyzed by immunohistochemical staining regarding the colocalization of viral infection, MHC class I expression and lymphocytic infiltrates. **(A)** Leucocyte common antigen was detected by immuno-alkaline phosphatase staining to visualize lymphocytes (red, cytoplasmic). **(B)** HCMV immediate early antigen (brown, nuclear, indicated by an open arrowhead) and MHC I molecules (red, cytoplasm and cell surface) were detected by immuno-peroxidase and immuno-alkaline phosphatase techniques, respectively. **(C)**. CD8 was detected by immuno-alkaline phosphatase staining to identify cytotoxic lymphocytes (red, cytoplasmic). Nuclei were counterstained with hematoxylin (blue). The scale bar is valid for all panels.

In order to get an idea of whether HCMV infection and inflammatory infiltrates are correlated and whether the effect of such infiltrations promotes or inhibits HCMV infection, we analyzed serial sections for the association between infected cells and leukocytes. The first section was immuno-alkaline phosphatase-stained for leucocyte common antigen (CD45), a pan-leukocyte marker expressed in cells of the myeloid and the lymphoid lineage (Figure 6A), the second section was stained by immunoperoxidase staining of immediate early antigen or by double labeling with MHC class I as described before (Figure 6B), and the third section was immuno-alkaline phosphatase-stained for CD8 as a marker for cytotoxic lymphocytes (Figure 6C).

**Figure 7:**
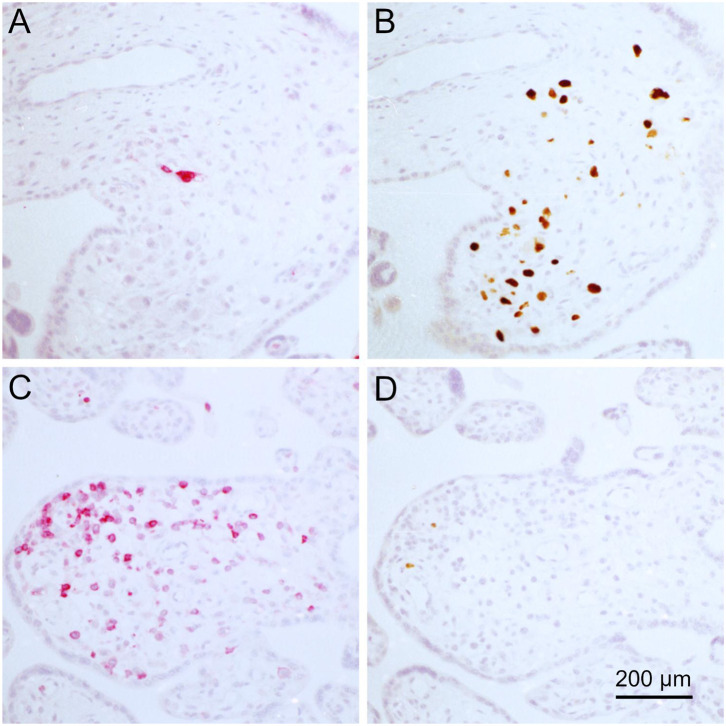
Negative correlation of infected cells and lymphocytes in placental tissue. Serial sections from HCMV-infected placenta tissue were analyzed by immunohistochemical staining regarding the colocalization of viral infection and lymphocytic infiltrates. **(A,C)** Leucocyte common antigen was detected by immuno-alkaline phosphatase staining to visualize lymphocytes (red, cytoplasmic). **(B,D)** HCMV immediate early antigen (brown, nuclear, indicated by an open arrowhead) was detected by immuno-peroxidase staining. Nuclei were counterstained with hematoxylin (blue). The scale bar is valid for all panels.

**Figure 8:**
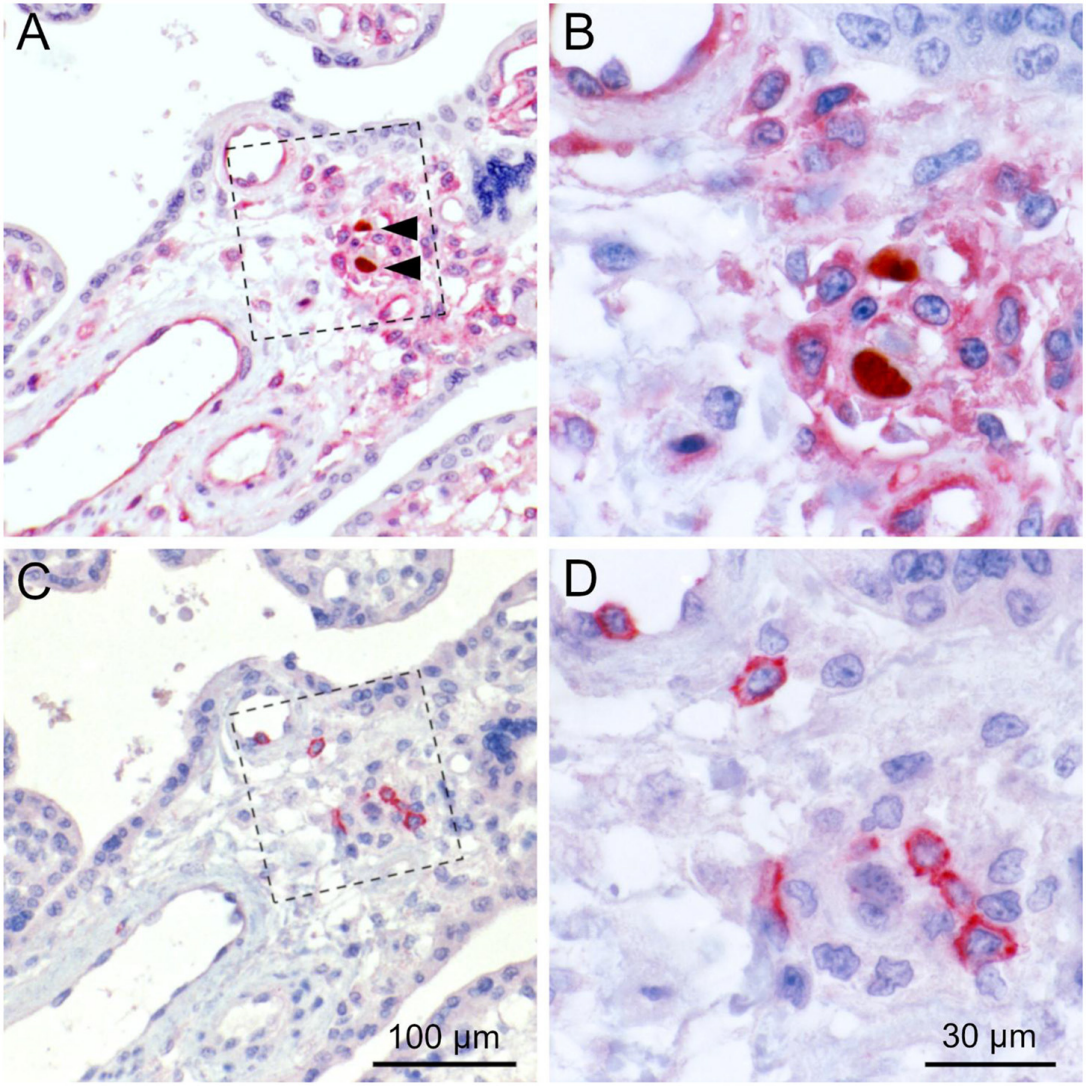
Colocalization of cytotoxic lymphocytes with infected cells. Serial sections from HCMV-infected placenta tissue were analyzed by immunohistochemical staining regarding the colocalization of viral infection, MHC class I expression and cytotoxic lymphocytes. **(A)** HCMV immediate early antigen (brown, nuclear, indicated by an open arrowhead) and MHC I molecules (red, cytoplasm and cell surface) were detected by immuno-peroxidase and immuno-alkaline phosphatase techniques, respectively. **(B)** Detail enlarged from **(A)** as indicated by the frame. **(C)** CD8 was detected by immuno-alkaline phosphatase staining to identify cytotoxic lymphocytes (red, cytoplasmic). **(D)** Detail enlarged from **(C)** as indicated by the frame. Nuclei were counterstained with hematoxylin (blue). Infected cells are indicated by filled arrowheads. The scale bars in **(C,D)** are also valid for **(A,B)**, respectively.

All villi that contained infected cells (total 159) were categorized for the degree of infection and the degree of inflammatory infiltration. Concerning infection, we distinguished single infected cells, several infected cells dispersed in the villus or clusters of three or more infected cells indicating focal spread. Leukocyte infiltrates were regarded weak if there were single leukocytes in the villus, medium if there were several leukocytes and strong if there were dense infiltrates present (Table 1). The chi-squared test suggested that the two variables are not independent (*p* = 0.045) with focal spread of HCMV being more probable in the absence of dense leukocyte infiltrations. An illustration of the extreme situations (unlimited viral spread in the presence of a weak leukocyte response; virus control in the presence of a dense leukocyte infiltrate) with individual staining of CD45 and HCMV immediate early antigen in serial sections is provided in Figure 7. Comparison with the distribution of CD8-specific signals in the third section showed that these infiltrations consisted mainly of CD8+ cells (Figures 6A,C). At a closer look, the analysis of corresponding sites in serial sections revealed cytotoxic T cells were often located in close contact to HCMV infected cells (Figure 8). Taken together these findings favored the idea that the presence of leukocytes had an antiviral effect.

**Table 1:** Contingency table and chi-squared test for the variables “leukocyte infiltration” and “HCMV infection” in villi of placental tissue.

**Observed frequencies**		**HCMV**			**Total**
		**Single**	**Dispersed**	**Focus**	
	Weak	18	9	28	55
LCA	Medium	40	25	24	89
	Strong	8	3	4	15
	Total	66	37	56	159
**Expected frequencies**		**HCMV**			**Total**
		**Single**	**Dispersed**	**Focus**	
	Weak	23	13	19	55
LCA	Medium	37	21	31	89
	Strong	6	3	5	15
	Total	66	37	56	159
**Observed/expected**		**HCMV**			***p*****-value**
		**Single**	**Dispersed**	**Focus**	
	Weak	0,8	0,7	1,4	
LCA	Medium	1,1	1,2	0,8	0,045
	Strong	1,3	0,9	0,8	

*LCA: weak, no or single cells; medium, several cells; strong, dense infiltrate. HCMV: focus, three or more infected cells in direct contact*.

## Discussion

This immunohistochemical analysis of selected cases of HCMV infection in different organs and conditions provides novel information on the balance between the virus and the host's immune response on the tissue level, which may also provide an idea of how HCMV manages to establish a lifelong persistence despite the remarkable frequency of CD8+ T cells that are directed against this virus. While reports on the viral immunoevasins US2, US3, US6, and US11 highlight the capability of HCMV to escape from cytotoxic T-cell responses, analyses of the host's T cell response emphasize the fact that HCMV can be well controlled by antigen-specific CTLs. Our findings can reconcile both notions as they show a remarkably strong MHC class I downmodulation in HCMV-infected cells but also infiltrates by CD8+ lymphocytes which obviously can limit viral spread in the tissue.

The simple fact that HCMV-infected cells in the late replication stage can be detected in tissues despite the presence of cytotoxic T-cells strongly suggests that they escaped antiviral responses at least for a few days, as this is the time frame HCMV needs to proceed to the late stage. Together with the findings that such late stage infected cells were typically mostly negative for MHC-class I signals and that cytomegalic cells, suggestive of being in the late stage, were often in direct contact with CTLs, it is tempting to imagine a scenario, in which MHC class I downmodulation actually allows late stage infected cells to withstand detection and lysis by CTLs and to continue producing viral progeny. On the other hand, given the finding that uninfected cells in the adjacent tissue strongly express MHC class I molecules, it is conceivable that transfer of virus to these cells might result in at least some degree of antigen presentation and control by the CTLs that have already been attracted to such sites. While we cannot formally prove that the CTLs in the surrounding of infected cells are HCMV-specific this is highly probable considering similar findings in the murine cytomegalovirus model under well-controlled conditions, where the epitope-specificity of the infiltrating lymphocytes has been demonstrated (Böhm et al., 2008a).

Due to its design, this study has certainly limitations and is meant as a “proof of principle” analysis and a starting point for future attempts that may investigate certain aspect in more detail. First, as retrospective immunohistochemical analyses of infected tissues are descriptive in nature, only statistical correlations can be detected between the analyzed variables, (i.e., infection, MHC class I expression, and lymphocytic infiltrates), but conclusions on a causal relationship between these factors will remain speculative. Yet, as controlled treatment with virus and virus-specific lymphocyte in varying concentrations and kinetics is not possible as HCMV is restricted to the human host, retrospective analysis of tissues from infected individuals is the best option that is available. Second, our analysis only visualizes the presence of MHC class I molecules but cannot evaluate their functionality. Hence, we can only address the effects of US2 and US11, which target MHC class I molecules for degradation, but not US3 and US 6, which block the translocation of peptide-loaded MHC complexes or translocation of peptides into the ER, respectively. And third, antibody HC10 detects a subset of MHC class I molecules including all HLA-B and HLA-C variants and a number of HLA-A variants and these were found clearly downmodulated. We cannot formally exclude that certain alleles of HLA-A that are not detected by this antibody behave different from the molecules that were visualized. However, given the overlapping effect of US2 and US11 on the various HLA-A alleles, it is unlikely that an additional visualization of those alleles would change the overall pattern found with HC10. Lastly, the low number of tissues analyzed and the lack of information regarding the HLA types of the respective tissues excludes any conclusions on whether HCMV downmodulation is a regular finding and whether the degree of downmodulation depends on the HLA alleles. Certainly, many important questions are still open, (e.g., the influence of HLA types or cell types on the degree of HCMV down modulation and the effect of MHC downmodulation on the degree of viral replication).

While the widely used MHC class I-specific antibody W6/32 is limited to use in frozen sections, antibody HC10 has the advantage that it can also be used in sections from formalin-fixed paraffin-embedded tissues (Stam et al., 1990; Mattijssen et al., 1991; Zhang et al., 2013). Unlike W6/32, which recognizes a conformational epitope shared among products of the HLA A, B, and C loci, HC10 has a more pronounced specificity for HLA-B and -C heavy chains and reacts only with certain of the HLA-A isoforms. Despite these slight differences in the specificity, however, the staining patterns of W6/32 and HC10 in frozen and paraffin-embedded sections, respectively, have been shown to correlate well (Mattijssen et al., 1991) and HC10 was successfully applied for analyses of MHC class I expression in formalin-fixed paraffin-embedded tissue sections. We cannot comment on HLA-G expression in trophoblast cells as this protein is not detected by HC10 (Gauster et al., 2007), but this does not detract too much from the relevance of our findings because trophoblasts are not a prominent target cell of HCMV in placental tissue (Sinzger et al., 1993), which was also the case in the tissue analyzed here. Due to these facts, the staining pattern seen with HC10 can be regarded as relevant for the detection of MHC class I downmodulation in HCMV-infected cells in the tissue sections that we investigated.

Our finding that depletion of MHC class I molecules in infected cells was most prominent beyond the immediate early stage of infection fits with initial reports on early expression kinetics of US2 and US11 and their mode of action, which results in degradation of MHC class I molecules in the early and late replication phase (Jones et al., 1995, 1996; Wiertz et al., 1996; Chambers et al., 1999). Our finding of cells in the immediate early stage that are lacking MHC class I signals may appear surprising in the context. However, reevaluation of the expression kinetics has revealed that US2 and US11 are also expressed under experimental immediate early conditions, indicating that they may be active at that stage under certain conditions (Hesse et al., 2013), and this was further corroborated by a comprehensive approach demonstrating US2, US3, and US11 at 6 h after infection at the surface of infected cells (Weekes et al., 2014). The complete lack of HC10 signals in many of the infected cells may appear surprising at a first glance because HLA-C has been reported to escape from downmodulation by HCMV (Schust et al., 1998; Barel et al., 2003) and successfully present antigen to HLA-C-restricted cytotoxic T cells (Ameres et al., 2013). Hence, it is tempting to speculate that late stage infected cells that appear empty in our HC10-staining nevertheless express some level of HLA-C, which may be sufficient to mediate recognition by T cells with the respective specificity. On the other hand, US6 has been shown to have the potential to induce degradation of HLA-C molecules (Jun et al., 2000).

Regarding the finding of elevated MHC class I expression at sites of HCMV infection, various explanations are conceivable. In principle, this could reflect that MHC class I high expressing cells are more susceptible to infection and hence infection is more pronounced at sites of higher MHC class I expression. However, this is highly unlikely, as no data are available in support of this explanation. It is more plausible to assume an immune reaction in response to infection with infiltrating lymphocytes (Böhm et al., 2008a) that can release interferons and other cytokines for upregulation of MHC class I molecules at sites of infection (Benz and Hengel, 2000). As a third explanation, infiltration of tissue by HCMV-infected monocytes can result in both HCMV infection and cytokines that upregulate MHC class I expression (Yurochko and Huang, 1999; Chan et al., 2008, 2012). In both cases, upregulated levels of MHC class I expression would lead to an alert state in the infected tissue facilitating recognition and control of newly infected cells by virus-specific cytotoxic lymphocytes.

The negative statistical correlation that we found in placental tissue between the density of infected cells and the density of infiltrating leukocytes suggests that the immune response had an antiviral effect in the respective tissue, as it is hard to imagine how less infection could cause larger infiltrations. Alternatively, a common cause could be responsible for both restriction of infection and attraction of leukocytes but this also appears less likely than a contribution of the infiltrates to restriction of viral spread. This explanation for the reciprocal correlation is supported by a previous finding in the murine CMV model that the presence of virus-specific CTLs restricted MCMV spread in liver tissue whereas a mutant lacking the respective epitope did not attract lymphocytes and formed large foci of infected cells (Böhm et al., 2008a).

Regarding the question of how specific CTLs can be induced despite the expression of viral immunoevasins that can block MHC class I-mediated antigen presentation, several explanations are possible. Cross presentation by professional antigen presenters like dendritic cells upon uptake of material from lysed infected cells may be central for priming of a CTL response (Arrode et al., 2000; Tabi et al., 2001; Weck et al., 2008; Snyder et al., 2010; Busche et al., 2013) and, paradoxically, downmodulation of MHC class I molecules in infected cells can even promote this response by increasing the amount of antigen in the tissues (Böhm et al., 2008b). Direct presentation may however also be crucial, [e.g., for memory inflation during phases of viral latency (Seckert et al., 2011)]. Importantly, escape of HCMV from antigen presentation by downmodulation of MHC class I molecules is probably never complete, but it can favor viral replication to some extent by restricting the number of effective epitopes and the time window of presentation to cytotoxic lymphocytes (Ameres et al., 2013), thus promoting a balance between virus and host that enables lifelong persistence of this virus.

## Data Availability Statement

All datasets generated for this study are included in the article/supplementary material.

## Ethics Statement

Ethics approval was not required for this study since tissue samples were provided by the Cooperative Human Tissue Network, a National Cancer Institute supported resource. Other investigators may have received samples from these same tissue specimens.

## Author Contributions

FG, GJ, DS, and CS designed the project. DS provided reagents. FG and CS performed experiments and evaluated the data and drafted the manuscript, which was critically revised by GJ and DS. All authors contributed to the article and approved the submitted version.

## Conflict of Interest

The authors declare that the research was conducted in the absence of any commercial or financial relationships that could be construed as a potential conflict of interest.
